# Chinese patent medicines for coronary microvascular disease: clinical evidence and potential mechanisms

**DOI:** 10.7150/ijms.85789

**Published:** 2023-06-04

**Authors:** Zhihua Yang, Yangxi Liu, Zhihui Song, Yujian Fan, Shanshan Lin, Zhao Ge, Shaoling Feng, Yu Liu, Yingfei Bi, Yi Wang, Xianliang Wang, Jingyuan Mao

**Affiliations:** 1 First Teaching Hospital of Tianjin University of Traditional Chinese Medicine, National Clinical Research Center for Chinese Medicine Acupuncture and Moxibustion, Tianjin, 300381, China.; 2 Institute of Traditional Chinese Medicine, Tianjin University of Traditional Chinese Medicine, Tianjin, 301617, China.

**Keywords:** coronary microvascular disease, traditional Chinese medicine, Chinese patent medicines, clinical evidence, potential mechanisms

## Abstract

Coronary microvascular disease (CMVD) is a high risk factor for many cardiovascular events. Due to the limited understanding of its pathophysiological mechanism, modern medicine still lacks therapeutic drugs for CMVD. Existing clinical studies have shown that traditional Chinese medicine (TCM) can effectively improve the clinical symptoms and quality of life of CMVD patients. As an indispensable part of TCM, Chinese patent medicines (CPMs) are widely used in clinical practice. In the face of numerous oral CPMs for treatment of CMVD, how to choose a reasonable medication regimen is one of the important issues in clinic. Based on this, this paper reviewed the clinical efficacy and recommended level of 12 CPMs in the treatment of CMVD, which are recommended by expert consensus on diagnosis and treatment of coronary microvascular disease with integrated Chinese and Western medicine (WM). In addition, this study also systematically summarized the possible mechanisms of CPMs in the treatment of CMVD by protecting coronary microvascular endothelial cells, improving vascular endothelial function, inhibiting inflammation, reducing oxidative stress, promoting angiogenesis, and improving hemorheology, aiming to provide meaningful information for its clinical application.

## Introduction

Coronary microvascular disease (CMVD) refers to a class of diseases characterized by subjective symptoms and/or objective evidence of myocardial ischemia due to insufficient myocardial blood supply and abnormal regulation of myocardial blood flow caused by the abnormal structure and/or function of coronary microvessels 1. There is no epidemiological information on CMVD in a large sample of people. A systematic review published in 2017 covering approximately 1.4 million subjects in 54 studies showed that the prevalence of CMVD without concomitant obstructive coronary artery disease (OCAD) was 67% in patients with stable angina, and it was considerably greater in women than in men 2. In 2020, a meta-analysis of 6,631 patients with non-obstructive myocardial ischemia using coronary flow reserve (CFR) to determine the presence of CMVD showed that compared with patients without CMVD, CMVD patients had a 3.93-fold increase in mortality and a 5.16-fold increase in the incidence of adverse cardiovascular events 3. Another meta-analysis that included 37 studies and 7212 patients with non-obstructive myocardial ischemia revealed that the prevalence of CMVD was about 41% and that female patients had a higher risk of developing CMVD compared to male patients 4. And patients with CMVD have a significantly increased risk of major adverse cardiovascular events (MACE) relative to the healthy population 5-8. Furthermore, CMVD has been associated with independently worsening diastolic dysfunction, and patients with diastolic dysfunction combined with CMVD had a 5-fold increased risk of hospitalization due to heart failure with preserved ejection fraction 5. Therefore, the diagnosis and treatment of CMVD is of great clinical importance.

In recent years, the understanding of CMVD is gradually deepening both domestically and internationally. In 2017, academician Zhang Yun and others developed the Chinese Expert Consensus on the Diagnosis and Treatment of CMVD 1, which is the first expert consensus on CMVD to be published both domestically and internationally. It has received significant attention from both domestic and international cardiovascular disease academia. Since then, seven guidelines/expert consensus regarding CMVD have been published in China and overseas. In 2018, the Coronary Vasomotion Disorders International Study Group (COVADIS) proposed international diagnostic criteria for type 1 microvascular abnormalities or primary CMVD and included slow flow on coronary angiography as one of the evidence of coronary artery microvascular abnormalities 9.In 2019, the European Society of Cardiology (ESC) classified CMVD as an important type of chronic coronary syndrome and proposed corresponding diagnostic and treatment strategies^10^. The American Heart Association (AHA) indicates that CMVD may be an important cause of myocardial infarction with no-obstructive coronary arteries (MINOCA)^11^. In 2020, the European Association of Percutaneous Cardiovascular Interventions (EAPCI) and the ESC jointly published a consensus document on Ischemia and No Obstructive Coronary Artery Disease (INOCA), proposing CMVD and/or subepicardial coronary artery spasm as the main cause of INOCA 12. The Expert Consensus on the Diagnosis and Treatment of Multidisciplinary Microvascular Illnesses in China, released by the Chinese Geriatrics Society in 2020^13^, covered the pathological characteristics and common prevention and control measures of multi-organ microvascular diseases. In 2022, Expert Consensus on the Diagnosis and Treatment of Coronary Artery Microvascular Diseases with Integrated Chinese and Western medicine (WM), jointly led by the Cardiovascular Committee of the China Association of TCM, Collateral Disease Committee of Beijing Society of traditional Chinese medicine (TCM) and Dongzhimen Hospital of Beijing University of TCM, was the first published expert consensus on the treatment of CMVD with proprietary TCM.

Currently, the progress of WM treatment of CMVD is relatively slow, and there is no drug available for CMVD. Clinical treatment of CMVD is still dominated by traditional cardiovascular drugs such as nicorandil, statins, calcium channel blockers and renin-angiotensin system blockers 14. There is no specific disease name for CMVD In TCM. Based on clinical symptoms, it can be classified under the category of "chest obstruction and heart pain" in TCM. TCM has many advantages, such as multi-components, multi-targets, multi-pathways, wide action mechanisms, minimal adverse effects, and good patient compliance, which play a unique role in the prevention and treatment of CMVD. The pathogenesis of CMVD is complex, and currently effective treatment methods are lacking. Under the guidance of the theory of holistic concept and the syndrome differentiation and treatment, individualized TCM treatments have shown good clinical efficacy in managing CMVD. In recent years, the research of TCM in the prevention and treatment of CMVD is deepening. Our team has previously conducted a review of clinical studies in the field of TCM against CMVD, and summarized 71 representative randomized controlled trials involving 34 oral Chinese patent medicines (CPMs), 11 TCM injections and 26 TCM decoctions. Our previous summary results showed that compared with the use of conventional WM alone, the combination of TCM can further alleviate angina symptoms in CMVD patients, improve the TCM evidence score and ECG ischemia, improve quality of life and exercise tolerance, improve coronary microcirculation disturbance, increase CFR, decrease the index of microvascular resistance (IMR), protect vascular endothelial function, inhibit inflammatory responses in the body, reduce blood lipids, improve long-term prognosis and reduce rehospitalization rate 14. Our preliminary summary of clinical evidence shows that TCM has great potential in the field of CMVD prevention and treatment.

CMVD is one of the dominant diseases in TCM treatment. CPMs is a modern preparation processed according to the prescribed prescription and preparation technology under the guidance of the TCM theory. Compared with TCM decoction, oral CPMs has the advantages of convenient to carry, controllable dose and good taste, and occupies an important position in the field of medical care in China. At present, there are few high-quality evidence-based clinical studies on the treatment of CMVD with CPMs that meet the requirements, and some outcome indicators have not been fully supported by evidence-based evidence. Moreover, there is a lack of evidence-based suggestions for the diagnosis and treatment of CMVD-integrated TCM and WM. In the face of many oral CPMs for treatment of CMVD, how to choose the reasonable drug regimen is one of the important clinical problems. The Expert Consensus on the Diagnosis and Treatment of CMVD with integrated Chinese and WM is the first expert consensus on the treatment of CMVD with proprietary TCM, and it is extremely important for increasing the efficacy of CMVD treatment by serving as a guide and reference for TCM and WM specialists, general practitioners and primary care physicians 15. This paper systematically summarizes and analyzes the clinical evidence and pharmacological mechanisms of action of CPMs recommended by Expert Consensus on the Diagnosis and Treatment of CMVD with integrated Chinese and WM for the treatment of CMVD and provides a scientific basis for their clinical application.

## Classification and diagnostic criteria of CMVD

### Classification of CMVD

According to the different etiologies of CMVD, it can be divided into three categories: CMVD without OCAD, CMVD with OCAD, and other types of CMVD 1. CMVD without OCAD includes microvascular angina and coronary slow flow phenomenon 15. There are three common clinical types of CMVD with OCAD: 1) patients with stable angina pectoris and coronary artery disease (CAD) heavier than the symptoms expected from the degree of coronary stenosis; 2) acute coronary syndrome; 3) after emergency percutaneous coronary intervention (PCI), the blood vessels were re-routed, but the myocardial perfusion was not restored, that is, there was no reflow^15^.

### Diagnostic criteria of CMVD

#### CMVD without OCAD

Referring to the 2020 EAPCI Expert Consensus Document on INOCA 12, angina symptoms with objective evidence of myocardial ischemia should be satisfied, but coronary angiography or coronary computed tomography angiography (CTA) showed no subepicardial OCAD, as seen in Table 1.

#### CMVD with OCAD

According to the Chinese Expert Consensus on the Diagnosis and Treatment of CMVD^1^, CAD patients can be diagnosed as CMVD complicated with OCAD if one of the following five conditions occurs after revascularization to relieve epicardial coronary artery stenosis: 1) CFR < 2.0 is measured after intra-coronary injection of adenosine or dipyridamole; 2) There is no spasm in the subepicardial coronary artery after intra-coronary injection of acetylcholine, but typical angina pectoris and ischemic ST-T changes in electrocardiogram are observed; 3) Coronary angiography criminal vascular thrombolysis in myocardial infarction (TIMI) blood grading is 0~2; 4) SPECT shows no local perfusion area of myocardium before discharge; 5) CMR imaging shows myocardial perfusion defect or gadolinium delayed enhancement.

## TCM treatment of CMVD

### TCM syndrome diagnosis of CMVD

CMVD belongs to the category of TCM “chest obstruction” and “heartache”, which can refer to TCM “chest obstruction” and “heartache” for syndrome differentiation and treatment. The common TCM syndromes of CMVD include blood stasis, phlegm turbidity, qi deficiency, qi stagnation, Yin deficiency, and Yang deficiency. The common complex syndrome type is qi deficiency and blood stasis syndrome, followed by phlegm and blood stasis syndrome, Yang deficiency and blood stasis syndrome, and qi stagnation and blood stasis syndrome 15. The main clinical symptoms of CMVD are paroxysmal chest tightness or chest pain, which can be divided into 6 kinds of syndromes. TCM syndrome differentiation of CMVD is seen in Table 2.

### Clinical evidence of CPMs treatment of CMVD

Search strategy/methodology: According to the inclusion criteria, literature was retrieved from CNKI, Wanfang Data, VIP Journal database, PubMed, the Cochrane Library, EMbase and other databases from the establishment of the database to May 2022, and supplementary retrieval was conducted by referring to the literature included in the systematic reviews included in this study. The search terms used to define the disease included: coronary microvascular disease, coronary microvascular dysfunction (CMD), microvascular angina, microvascular chest pain, cardiac syndrome X, coronary slow flow phenomenon, coronary slow flow, no-reflow.

Inclusion criteria: Subjects: Patients with CMVD, including CMD, microvascular angina (cardiac syndrome X), coronary slow flow phenomenon, and no reflow after PCI. Intervention and control measures: ① The experimental group was treated with a Chinese patent medicine or external treatment of TCM combined with conventional WM, the control group was treated with conventional WM; ② The experimental group was treated with a Chinese patent medicine or external treatment of TCM, the control group was treated with placebo or routine nursing measures. Type of study: Randomized controlled trials (RCTs) or systematic reviews.

Exclusion criteria: ①Studies with missing important data such as sample size and outcome measures; ② Studies lack comparability of baseline data between groups.; ③ Repeatedly published studies; ④ Studies for which full-text reports could not be obtained; ⑤ Studies that significantly violate the diagnostic and treatment routine.The clinical evidence for this consensus was constructed by searching the currently published RCTs or systematic reviews of the treatment of CMVD with CPMs.

The Expert Consensus on the Diagnosis and Treatment of CMVD with integrated Chinese and WM recommends 12 CPMs for the treatment of CMVD, among which the majority are for the treatment of qi deficiency and blood stasis syndrome, and qi stagnation and blood stasis syndrome, while the treatment of other syndromes of CMVD is insufficient, as shown in Table 3,^15^. Based on Table 3, the clinical evidence of CPMs for CMVD is summarized in Table 4.

## Microvascular angina

### Qi deficiency and blood stasis syndrome

Syndrome differentiation of microvascular angina belongs to qi deficiency and blood stasis syndrome except for the typical symptoms of angina pectoris, there is also shortness of breath and fatigue, and the symptoms are aggravated during activity. Based on the routine treatment of WM, Tongxinluo capsule, Qishen Yiqi dropping pills or Naoxintong capsule (strong recommendation) and Shexiang Tongxin dripping Pills (weak recommendation) can be used for such patients 16. Existing clinical studies have shown that the combined use of Tongxinluo capsule or Naoxintong capsule based on the routine treatment of WM can reduce the attack times of angina pectoris 16-18, shorten the duration of each angina pectoris attack 18, prolong the duration of treadmill exercise 19, 20, and reduce the dosage of nitroglycerin 18. Compared with the routine treatment of WM, Qishen Yiqi dropping pills combined with routine WM can decrease IMR in patients with microvascular angina pectoris. For patients with heart failure, Qishen Yiqi dropping pills can increase left ventricular ejection fraction (LVEF) and improve cardiac function 21. A meta-analysis of randomized controlled trials (RCTs) including 3537 patients after PCI showed that the application of Qishen Yiqi dripping Pills combined with WM in the treatment of patients after PCI could reduce the occurrence of major adverse cardiovascular events (MACE), improve the clinical efficacy, quality of life and prognosis compared with WM treatment alone 22. Shexiang Tongxin dripping pills by themselves can decrease IMR in patients with microvascular angina as compared to the untreated group. Compared with the routine treatment of WM alone 23, Shexiang Tongxin dripping pills combined with routine treatment of WM can prolong the duration of patients' treadmill exercise and reduce the frequency of angina pectoris attacks 24. A meta-analysis demonstrated that Shexiang Tongxin dropping pills combined with conventional therapy of CAD was more effective than conventional therapy in the indicators of the total effective rate, electrocardiogram efficacy, the number and duration of angina attack, and improve blood lipids 25.

### Qi stagnation and blood stasis syndrome

Syndrome differentiation of microvascular angina belongs to qi stagnation and blood stasis syndrome except for typical angina pectoris, at the same time, it can be seen that the pain in the chest and flank is aggravated with the change of mood. Based on the routine treatment of WM, such patients can be treated with Shexiang Baoxin pills, Compound Danshen dripping pills, Wide chest aerosol, Suxiao Jiuxin pills, Xuefu Zhuyu capsule (strong recommendation), and Yindan Xinnaotong soft capsule (weak recommendation). Shexiang Baoxin pills 26-28, Compound Danshen dripping pills 29, Suxiao Jiuxin pills 30 or Yindan Xinnaotong soft capsule 31 combined with WM in the treatment of patients with microvascular angina, compared with the routine treatment of WM, it can reduce the frequency of angina pectoris attacks. A multicenter randomized double-blind placebo-controlled trial including 200 acute coronary syndrome (ACS) with early PCI patients showed that Suxiao Jiuxin pills (SJP) with WM was more effective than WM treatment alone in the indicators of improvement of heart function and quality of life, lowed incidence of MACE, increased score in the seattle angina questionnaire (SAQ) subscale and decreased the serum concentration of fibrinogen (Fib) and cystatin C (cysC)^32^. Compared with the routine treatment of WM, the combination of Shexiang Baoxin pills 33-35 or Yindan Xinnaotong soft capsule 36 can decrease IMR. A Meta-Analysis analysis results demonstrated that Yindanxinnaotong soft capsule could regulate coagulation condition and fibrinolytic system in patients with CAD and improve myocardial ischemia, angina symptoms, and heart function, reduce restenosis after PCI, and regulate blood lipids, control the long-term development of the cardiovascular disease and prevent the cardiovascular events from happening again 37. Shexiang Baoxin pills 38 or Xuefu Zhuyu capsule 39 combined with routine WM can prolong the duration of treadmill exercise in patients with microvascular angina. A systematic review and meta-analysis analysis results showed that Shexiang Baoxin pills could demonstrate a beneficial role in patients with CAD after PCI of reducing the incidence of MACE and improving LVEF, N-terminal pro-B-type natriuretic peptide (NT-pro-BNP), inflammatory mediators, and blood lipid index 40. Wide chest aerosol can reduce the number of corrected TIMI frame grading during coronary angiography in patients with microvascular angina 41. A non-inferiority multi-center RCT demonstrated that Wide chest aerosol can rapidly relieve angina pectoris. Furthermore, Wide chest aerosol has superior effect, and better tolerability than nitroglycerin tablet among Canadian Cardiovascular Society Ⅱ and Ⅲ patients 42.

### Qi and Yin deficiency syndrome

The syndrome differentiation of microvascular angina belongs to the qi and Yin deficiency syndrome. In addition to the typical symptoms of angina pectoris, there are also symptoms of qi deficiency such as fatigue, shortness of breath, aggravation of labor, and Yin deficiency such as tidal fever and sweating, night sweats, and red tongue with little coating. Such patients can be treated with Tongmai Yangxin pills based on conventional WM (strong recommendation). Compared with conventional WM, Tongmai Yangxin pills combined with conventional WM can reduce the positive rate of treadmill exercise for patients with microvascular angina 43. A systematic review of RCTs showed that compared with the single application of WM treatment, the combined administration with Tongmai Yangxin pills and WM has a better clinical efficacy in the treatment of angina pectoris of CAD in terms of the improvement rate of electrocardiogram and the clinical efficacy of TCM syndrome 44. Modern pharmacological research indicated that Tongmai Yangxin pills could improve the cardiac structure and function, reduce muscle fiber swelling and inflammatory cell infiltration, alleviate cardiomyocyte injury, and significantly reduce myocardial ischemia reperfusion with no-reflow 45.

### Heart blood stasis syndrome

The differentiation of microvascular angina pectoris belongs to the heart blood stasis syndrome with clinical manifestations of chest pain, chest tightness, and lip and/or tongue dark purple can be treated with CPMs for activating blood and resolving stasis, dredging collaterals and relieving pain such as Danshen tablets (weak recommendation). Danshen tablets combined with conventional WM can prolong the duration of treadmill exercise, and reduce the frequency of angina attacks and the dosage of nitroglycerin in the treatment of microvascular angina patients compared with conventional WM 46.

### ST-elevation myocardial infarction

For patients needing revascularization, the application of TCM intervention based on conventional WM treatment before surgery can reduce the incidence of patients with no reflow after PCI. Clinical evidence shows that Tongxinluo capsule combined with conventional WM can be used to treat acute myocardial infarction patients and reduce the no-reflow rate (strong recommendation). For patients with ST-elevation acute myocardial infarction (STEMI), combined application of Tongxinluo capsule and conventional WM before emergency PCI further reduced the no-reflow rate within 24h compared with conventional WM 47. A randomized double-blind placebo-controlled study including 3652 patients with CAD after PCI provided evidence that, compared with conventional treatment alone, Tongxinluo capsule as an adjuvant therapy had better clinical efficacy in the treatment of CAD after PCI and could effectively reduce the risk of angiographic restenosis, heart failure, angina, myocardial infarction, revascularization, and lower all-cause mortality and mortality due to any cardiovascular event on the 6-month course^48^.

### No-reflow after PCI

In the case of patients with no-reflow after PCI, combined with TCM intervention based on conventional WM treatment can further reduce the no-reflow rate of patients. Clinical studies have shown that Compound Danshen dripping pills combined with conventional WM can be used to improve coronary blood flow in patients with coronary no-reflow after PCI (strong recommendation). For patients with coronary no-reflow after PCI, compared with conventional WM, combined application of Compound Danshen dripping pills based on conventional WM after surgery can reduce the incidence of no-reflow 49. A meta-analysis of RCTs including twenty RCTs involving 2574 participants with CAD indicated that Compound Danshen dripping pills combined with PCI treatment prominently reduced the incidence of MACE, prominently improved vascular endothelial function and cardiac function, dramatically mitigated hemorheology, blood lipid parameters and inflammatory mediators compared with PCI alone 50.

In conclusion, current clinical studies suggest that CPMs can improve IMR and LVEF in CMVD patients, reduce the frequency of angina attacks, shorten the duration of angina, extend the time of treadmill exercise, reduce the dosage of nitroglycerin, improve activity tolerance and quality of life.

## Mechanism of action

**Figure 1:**
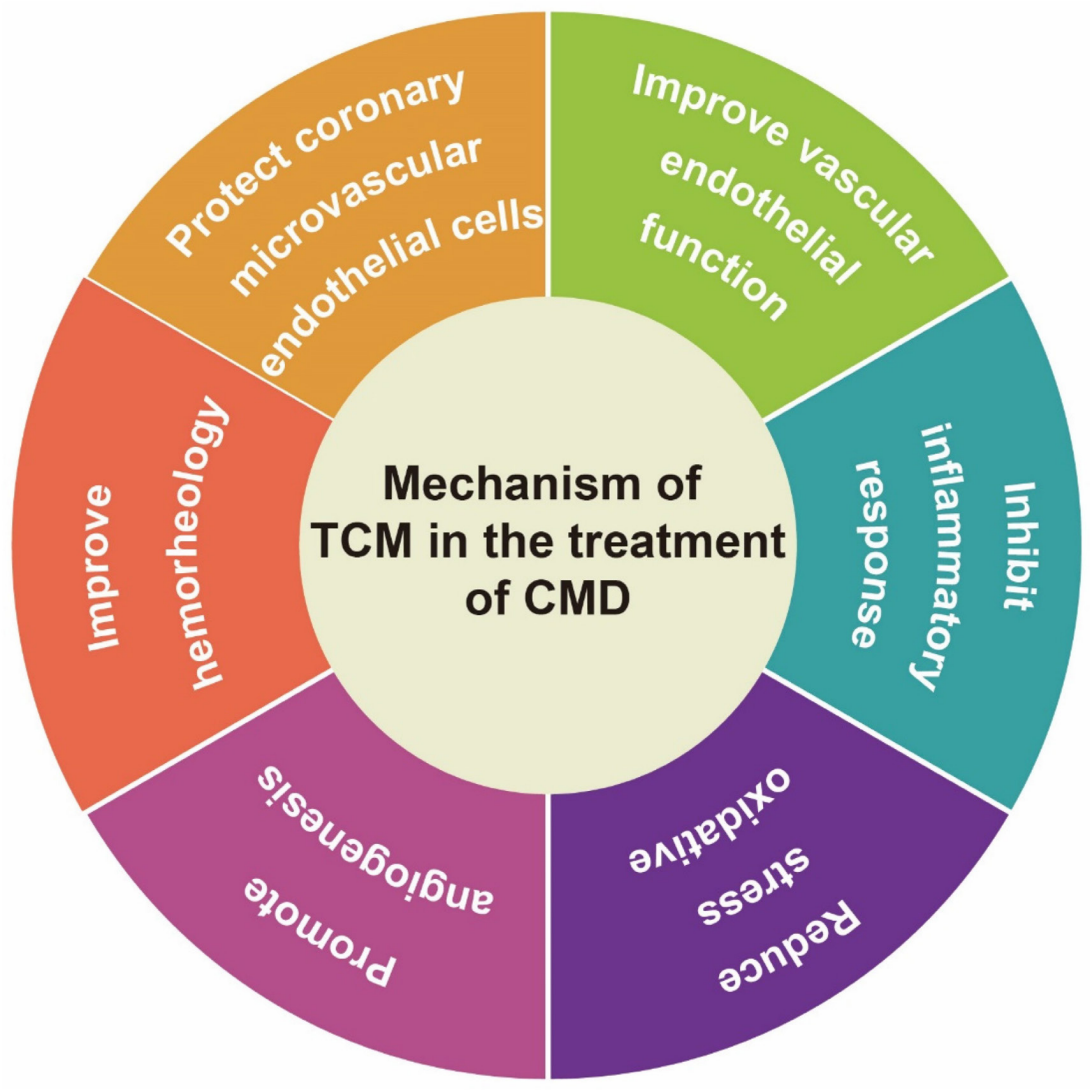
Mechanism of TCM in the treatment of CMD

CMVD is caused by the interaction of multiple factors and mechanisms, including abnormal microvascular function, vascular endothelial dysfunction, inflammatory response, oxidative stress, etc. These physiological and pathological changes play different roles in the occurrence and development of coronary microcirculation 12, 51. Existing studies have shown that TCM can treat CMVD by protecting coronary microvascular endothelial cells, improving vascular endothelial function, inhibiting inflammatory response, alleviating oxidative stress, promoting angiogenesis, and improving blood rheology (Figure 1).

### Protect coronary microvascular endothelial cells

Cardiac microvascular endothelial cells (CMECs) play an important role in maintaining the normal function of cardiac microvascular, and the dysfunction of CMEC is often preceded by myocardial injury 52. In the process of occurrence and development of CMVD, the injury of CMECs is the core link. The normal functions such as proliferation, adhesion, migration, apoptosis, and secretion of CMECs are damaged, leading to CMVD, which can cause coronary microvascular constriction, decreased CFR, and insufficient myocardial blood supply clinically, which are the early manifestations of CMVD 53, 54. An animal experiment has shown that Tongxinluo capsule can effectively protect the structure and function of myocardial microvascular endothelial cells after myocardial ischemia-reperfusion injury, protect microvessels and myocardial perfusion units, and reduce the size of no-reflow and myocardial infarction 55. Tongxinluo could protect Human cardiac microvascular endothelial cells (HCMECs) from hypoxia /reoxygenation (H/R) injury by promoting autophagy through activation of the mitogen-activated protein kinase/ERK pathway 56. Han et al. 57 the study found that Qishen Yiqi dropping pills could protect HCMECs from impairment by hypoxia or reoxygenation, in addition, Qishen Yiqi dropping pills could exhibit effects on attenuating microvascular damage and albumin leakage after ischemia/reperfusion (I/R) injury, showing a role in maintaining endothelial junctions, caveolae, and collagen in the basement membrane of microvessels.

### Improve vascular endothelial function

The vascular endothelium plays an important role in maintaining the normal vascular system. The abnormal coronary vascular endothelial function could cause the occurrence and development of a variety of cardiovascular diseases, thus protecting vascular endothelial function is of great significance for the prevention and treatment of CMVD. Coronary endothelial dysfunction is closely related to the imbalance between vasoconstrictor factor endothelin-1 (ET-1) and diastolic factor nitric oxide (NO) caused by vascular endothelial injury 58. Decreased production and excessive depletion of NO have been confirmed to be closely related to microvessels relaxation dysfunction, and enhanced vasoconstricttion function is associated with increased levels of ET-1, catecholamine, and acetylcholine. The signaling pathway mediated by NO is a protective factor of endothelial cells and can inhibit platelet activation by reducing the expression of cell adhesion markers and enhancing the activity of the cyclic guanosine monophosphate signaling pathway to reduce endothelial cell damage 59. Many studies have confirmed the improving effect of TCM on vascular endothelial function from the perspective of regulating the content of ET-1 and NO antagonistic factors, to treat CMVD. Tongxinluo capsule can improve the endothelial-dependent vasodilation mediated by brachial artery flow, increase the serum levels of NO and adiponectin, reduce the serum levels of ET-1, protect the vascular endothelial function in patients with CMVD, and then relieve the clinical symptoms of patients with angina pectoris 19, 60. Animal experimental studies have shown that Tongxinluo capsule can up-regulate the expression of related endothelial cell adhesion proteins and the activity of eNOS during reperfusion, reverse the hypertonic state of endothelial cells, improve the damage of microvascular endothelial functional barrier, and prevent the continuous development of myocardial reperfusion injury 61. Dong et al.'s study 62 found that Compound Danshen dripping pills combined with ticagrelor could significantly improve the levels of serum VEGF and NO, reduce the level of ET-1, improve vascular endothelial function in patients with angina after PCI, and thus reduce the number of angina attacks, shorten the duration of each angina, and improve the quality of life. Shexiang Baoxin pills can significantly reduce the frequency of angina attacks in CMVD patients, improve endothelial-dependent flow-mediated vasodilation (FMD) mediated by brachial artery flow, reduce serum Ang II, CRP, and ET-1 levels, increase serum NO level, and protect vascular endothelial function 28, 63.

### Inhibit inflammation

Coronary microvascular endothelial cell injury is the core of CMVD induction. Inflammatory response plays an important role in the occurrence and development of coronary microvascular endothelial dysfunction. Inflammatory factors not only cause vascular endothelial injury and intima thickening but also reduce the synthesis of NO and prostacyclin in endothelial cells, activating immune cells to release human endothelin (ET) and ET immune-like activators, resulting in abnormal endothelial function 64. A variety of inflammatory factors can participate in the occurrence and development of CMVD through the endothelial cell injury pathway 65, 66. Serum C-reactive protein (CRP) is a recognized marker of inflammation, and its serum level is often used to describe chronic low-grade inflammation in the whole body 67. CFR is an effective indicator of abnormal coronary microcirculation function when coronary angiography does not show obvious stenosis. In a study involving 21 CMVD patients and 21 healthy volunteers, Recio-Mayoral et al. 68 divided patients with CMVD into high CRP group (> 3mg/L) and low CRP group (≤3mg/L) according to CRP levels. The results showed that the CFR of the low CRP group was not significantly different from that of the healthy group, while the CFR of the high CRP group was significantly lower than that of the low CRP group. This study was the first to confirm the correlation between chronic low-grade inflammation and CMVD, and the effect of CRP on CFR was dose-dependent. Tong et al. 69 and Aryan et al. 70 found that hs-CRP-mediated chronic inflammation was an independent predictor of CMD in patients with ischemic heart disease. TNF-α, IL-6, and leptin can activate nicotinamide adenine dinucleotide phosphate oxidase in the vessel wall, which can reduce NO bioavailability by promoting hyperoxide anion production, ultimately leading to coronary microvessel diastolic function injury 71. Chen et al. 72 revealed that routine treatment combined with Danhong injection could reduce plasma levels of hypersensitive C-reactive protein (hs-CRP) and homocysteine, thus improving vascular endothelial function and relieving clinical symptoms of patients. Tongxinluo capsule can effectively reduce the levels of serum TNF-α, IL-1, and IL-6 in CMVD patients, increase the level of serum IL-10, inhibit the body's inflammatory response, and thus improve the clinical symptoms of patients 19. Shexiang Baoxin pills combined with conventional WM have a significant effect on patients with angina pectoris of CAD, which can effectively reduce the levels of IL-6 and hs-CRP and inhibit the inflammatory response of patients 33. Xuefu Zhuyu decoction, compound Danshen dripping pills, and Yindan Xinnaotong soft capsule can effectively improve the clinical symptoms of angina patients, and reduce the levels of serum inflammatory factors hs-CRP, IL-6, IL-1β, and TNF-α 36, 73, 74.

### Reduce oxidative stress

CMVD is caused by the interaction of multiple factors and multiple mechanisms, among which oxidative stress is considered to be one of the main mechanisms. Under oxidative stress, the production of oxygen free radicals increases, triggering mitochondrial dysfunction, calcium overload, DNA damage, and myocardial membrane lipid peroxidation, which can eventually lead to cell death 75. A large amount of ROS is produced during cell ischemia and hypoxia. When it exceeds the antioxidant capacity of the body, it can lead to lipid peroxidation of vascular endothelial cells, gradually peeling away from blood vessels, resulting in exposed collagen fibers under the endothelium, activating the endogenous coagulation system, and further causing platelet activation, up-regulation of adhesion factors, fibrin aggregation, a decrease of plasmin activity, and formation of microthrombus. These changes aggravate local ischemia and form a vicious cycle, resulting in CMVD 76. Yindan Xinnaotong soft capsule after acute myocardial infarction (AMI) emergency PCI can significantly reduce the level of malondialdehyde (MDA) and lipid peroxide (LPO), and increase the superoxide dismutase (SOD) level. The oxidative stress index of the body can be effectively improved, the oxidative stress injury can be alleviated, and then the myocardial perfusion of patients can be improved and the coronary blood flow reserve function can be enhanced 77. Tongxinluo capsule combined with routine WM treatment in patients with no-reflow after PCI can reduce serum MDA level, increase SOD level, inhibit oxidative stress, improve myocardial microperfusion, and improve cardiac function indicators, and thus improve the prognosis of patients 78. Gu et al. 73 clinical research found that Compound Danshen dripping pills could reduce the serum levels of LPO, SOD, and MDA in patients with angina pectoris of CAD, reduce the oxidative stress damage of the body, and improve the clinical symptoms of angina pectoris patients. Based on conventional WM treatment, Qishen Yiqi dripping pills can further reduce serum SOD, advanced glycation end products, MDA, advanced oxidation protein products oxidative stress indexes, reduce the oxidative stress injury of the body, and then relieve the clinical symptoms of myocardial ischemia, chest tightness, palpation and so on in CAD patients, improve the prognosis of patients 79.

### Promote angiogenesis

The decreased number of myocardial capillaries is one of the important determinants of CFR. Angiogenesis can effectively improve CMVD by increasing blood supply capacity, restoring cardiac function, and improving hemodynamics. Zhang et al. 80 found that Xuefu Zhuyu decoction could up-regulate the expression of vascular endothelial growth factor (VEGF) protein, increase the number of endothelial cells in ischemic myocardium, increase myocardial microvascular density, and promote angiogenesis in rats with acute myocardial ischemia. Chen et al. 81 found that Shexiang Baoxin pills promoted angiogenesis by up-regulating VEGF level in CAD patients. Clinical studies have found that based on conventional WM treatment, the combined application of Shexiang Baoxin pills can effectively reduce the levels of related hemorheological indexes such as fibrin, plasma viscosity, and whole blood viscosity in CAD patients, improve the levels of serum angiogenesis factors NO, NOS and VEGF, and further promote the establishment of coronary collateral circulation, reduce the incidence frequency and shorten the duration of CAD^82^. Zhang et al. 83 found that Shexiang Baoxin pills regulated endothelial cell function and signal transduction pathway by activating macrophages, promoting human umbilical vein endothelial cell proliferation and migration and tubule formation, angiogenesis factor mRNA and protein expression, and promoted angiogenesis. Shexiang Tongxin dropping pills clould alleviate cardiac dysfunction in rats with CMD caused by ligation of the left anterior descending branch by inducing M2 macrophage polarization to promote angiogenesis in ischemic myocardium 84. Vascular growth factors such as VEGF and basic fibroblast growth factor (bFGF) are helpful to increase vascular permeability, angiogenesis and intravascular homeostasis. Zhang et al. 85 found that Qishen Yiqi dripping pills could increase the vascular density of the left ventricle and reduce the infarct area by increasing the protein expression levels of VEGF and bFGF and mRNA level of platelet-derived growth factor-B in myocardial tissue of myocardial infarction rats.

### Improve hemorheology

Sustained hemodynamic abnormalities can lead to structural damage of microvessels, increased vascular walls permeability, capillary leakage, basement membrane damage and secondary thickening, vascular cavity stenosis or even occlusion, microthrombus formation, microcirculation ischemia, hypoxia, and then lead to organ dysfunction. The hemorheological parameters are closely related to the severity of CAD, and the improvement of various hemorheological parameters such as high viscosity can improve coronary blood flow. Li et al. 86 observed the influence of Qishen Yiqi dropping pills on hemorheology in elderly cardiovascular and cerebrovascular diseases patients, and confirmed that oral administration of Qishen Yiqi dropping pills based on the treatment of the primary disease could significantly reduce hemorheology indexes such as whole blood low shear viscosity, whole blood high shear viscosity, plasma viscosity, and hematocrit, and it is safe and effective. Huang et al. 87 studied the effects of the Compound Danshen dripping pills on hemorheology, blood lipid, and inflammatory response transmitters in elderly CAD patients. The results showed that Compound Danshen dropping pills combined with conventional WM could not only regulate lipid metabolism and reduce the inflammatory reaction, but also improve hemorheological indexes such as whole blood high shear viscosity, whole blood low shear viscosity, plasma viscosity, hematocrit, and erythrocyte sedimentation rate (ESR), and the efficacy is better than that of conventional WM alone. Xue et al. 63 found that Shexiang Baoxin pills combined with Nicodil could significantly improve cardiac function and hemodynamic indexes such as whole blood viscosity (high shear value, low shear value), plasma specific viscosity, and platelet adhesion rate in patients with coronary microcirculation dysfunction in myocardial infarction. Compared with WM alone, Xuefu Zhuyu decoction on the basis of conventional WM treatment can significantly improve the hemodynamics, reduce myocardial ischemia and improve clinical symptoms such as angina 74.

## Conclusion and perspectives

With the rapid development of evidence-based medicine and coronary interventional techniques, the clinical significance of CMVD has received increasing attention. CMVD has a complex etiology and pathogenesis, its treatment remains limited to improving lifestyle, controlling the traditional risk factors for atherosclerosis, and relieving myocardial ischemia. There are currently no large sample randomized clinical trials using cardiovascular events as the assessed endpoint and CMVD as the study subject. Therefore, it is unclear which therapy option can lower the rate of cardiovascular events in CMVD. And modern medicine still lacks an effective treatment for CMVD.

In recent years, the understanding of CMVD in TCM has been considerably advanced, and numerous clinical types of research have been conducted. The available clinical evidence suggests that treatment for CMVD with TCM has been shown to reduce the attack times of angina pectoris, shorten the duration of angina pectoris attacks, prolong the duration of treadmill exercise, reduce the dosage of nitroglycerin, ameliorate TCM syndrome, improve cardiac function, improve exercise tolerance and quality of life, reduce mortality and rehospitalization rates, and improve long-term prognosis, etc. But there is a lack of evidence-based suggestions for the Diagnosis and Treatment of CMVD with integrated Chinese and WM. This paper systematically summarizes the clinical evidence of proprietary TCM for the treatment of coronary microvascular disease recommended by the Expert Consensus on the Diagnosis and Treatment of CMVD with integrated Chinese and WM, however, few high-quality evidence-based clinical studies fit the criteria, and several of the outcome indicators are not yet supported by sufficient evidence-based evidence. The ultimate level of evidence and the severity of the recommendation were impacted by some of the included studies' confusing statements of sample size estimation, random assignment concealment strategies, blinded designs, and shedding rates. Future clinical studies on CMVD treatment with TCM should be conducted in large-sample, multicenter randomized clinical trials with cardiovascular events as the observed endpoint, to test the possibility of developing new drugs and therapies specifically targeting CMVD to improve clinical endpoints and to establish treatments with evidence-based medical evidence. TCM has multi-target and multi-path therapeutic characteristics, and certain results have been achieved so far, as research advances, it is thought that TCM offers enormous potential for CMVD prevention and therapy.

## Figures and Tables

**Table 1:** Diagnostic criteria of CMVD without OCAD

Diagnostic criteria of CMVD without OCAD		
Diagnostic criteria	Clinical evidence	Detailed parameters
1	Myocardial ischemia symptom	Effort or resting angina symptoms
2	Non-OCAD	Coronary angiography: Coronary stenosis < 50% or FFR > 0.80 Coronary artery CTA suggests no or mild stenosis
3	Objective evidence of myocardial ischemia	Ischemic electrocardiogram changes occur during angina attacks. Transient but reversible local wall motion abnormalities or perfusion defects were observed during SPECT/PET/ cardiac MRI/ echocardiography
4	Evidence of impaired coronary microvascular function	CFR < 2.0 and/or IMR ≥ 25

Note: CMVD without OCAD can be diagnosed by meeting 1-4 diagnostic criteria. CMVD: Coronary microvascular disease; OCAD: obstructive coronary artery disease; FFR: fractional flow reserve; SPECT: single-photon emission computed tomography; MRI: magnetic resonance imaging.

**Table 2:** Simple TCM syndrome differentiation of CMVD

Main symptom	Syndrome	Clinical manifestation
Paroxysmal chest tightness or chest pain	Blood stasis syndrome	Chest pain, pain on the shoulder and back, dark purple tongue, or petechiae, thin and white tongue coating, thin and unsmooth or intermittent pulse, accompanied by palpitation, palpitation, and so on. The main clinical features are chest pain/chest tightness, lip and/or tongue are dark purple
Paroxysmal chest tightness or chest pain	Qi deficiency syndrome	Palpitation, spontaneous sweating, fatigue, chest tightness and shortness of breath, aggravation of labor, light tongue, weak pulse, or intermittent pulse. The main clinical features are shortness of breath, fatigue, god exhausted and laziness in speech, and aggravation of labor
Paroxysmal chest tightness or chest pain	Qi stagnation syndrome	Chest and hypochondrium distension pain, sometimes stop, white tongue coating, pulse string or intermittent, and emotion-related. The main clinical features are chest and hypochondrium distension pain, shortness of breath, and emotion-related
Paroxysmal chest tightness or chest pain	Yin deficiency syndrome	Upset, dry mouth and pharynx, red tongue, less tongue coating, pulse thin and rapid or intermittent, accompanied by night sweats, vexing heart in the chest, palms, and soles. The main clinical features are tidal fever and sweating, night sweats, red tongue, and little tongue coating
Paroxysmal chest tightness or chest pain	Yang deficiency syndrome	Chest tightness, white face, cold limbs, weak or intermittent pulse, tongue fat with tooth marks. The main clinical features are chilly sensation and cold limbs, light fat tongue or tooth marks
Paroxysmal chest tightness or chest pain	Phlegm turbidity syndrome	Tongue coating thick and greasy, pulse string slippery or unsmooth, could be accompanied by chest and hypochondrium swelling and tightness, irregular menstruation, dysmenorrhea, body fat, and so on. The main clinical features are obesity and thick and greasy tongue coating.

Note: Patients may have multiple syndromes at the same time.

**Table 3:** Summary of recommendations for the treatment of CMVD with CPMs

Disease	Syndrome	Simple syndrome differentiation	Recommend CPMs	Recommendation rate	References		
Microvascular Angina	Qi deficiency and blood stasis	Typical angina symptoms + Fatigue, shortness of breath, aggravation of labor	Tongxinluo capsule	Strong recommendation	15		
			Qishen Yiqi dripping pills	Strong recommendation	15		
			Naoxintong capsule	Strong recommendation	15		
			Shexiang Tongxin dripping pills	Weak recommendation	15		
	Qi stagnation and blood stasis	Typical angina symptoms + Chest and hypochondrium distension pain, related to emotion	Shexiang Baoxin pills	Strong recommendation	15		
			Compound Danshen dripping pills	Strong recommendation	15		
			Wide chest aerosol	Strong recommendation	15		
			Suxiao Jiuxin pills	Strong recommendation	15		
			Xuefu Zhuyu capsule	Strong recommendation	15		
			Yindan Xinnaotong capsule	Weak recommendation	15		
	Deficiency of both qi and Yin	Typical angina symptoms +Qi deficiency symptoms + Tidal fever and sweating, night sweats, red tongue with little coating	Tongmai Yangxin pills	Strong recommendation	15		
	
	Heart blood stasis	Chest pain, chest tightness, the lip and/or tongue are dark purple	Danshen tablets	Weak recommendation	15		
STEMI		Typical myocardial infarction symptoms	Tongxinluo capsule	Strong recommendation	15		
No-reflow after PCI	Qi stagnation and blood stasis	Typical angina symptoms +Chest and hypochondrium distension pain, related to emotion	Compound Danshen dripping pills	Strong recommendation	15		

Note: Strong recommendation indicates that the advantages of the intervention clearly outweigh the disadvantages, or the disadvantages clearly outweigh the advantages, without strings attached. Weak recommendation indicates uncertainty regarding the balance between advantages and disadvantages, where evidence of varying quality shows comparable advantages and disadvantages; or conditional recommendation.

**Table 4:** Clinical evidence of TCM for CMVD

Chinese patent medicines	Compositions	Effect	Disease	Main finding	References
Tongxinluo capsule	Renshen (*Ginseng Radix Et Rhizoma*), Shuizhi (*Hirudo*), Quanxie (*Scorpio*), Chishao (*Paeoniae Radix Rubra*), Chantui (*Cicadae Periostracum*), Tubiechong (*Eupolyphaga Steleophaga*), Wugong (*Scolopendra*), Tanxiang (*Santali Albi Lignum*), Jiangxiang (*Dalbergiae Odoriferae Lignum*), Ruxiang (*Olibanum*), Suanzaoren (*Ziziphi Spinosae Semen*), Bingpian (*Borneolum*)	replenishing qi and activating blood, dredging collaterals and relieving pain	Microvascular angina	①Reduce the frequency of angina pectoris, shorten the duration of angina pectoris②Improve the total effective rate of clinical efficacy③Increase NO level and reduce ET-1 evel④Extend the total treadmill exercise times, enhance the time of ST-segment depression of 1 mm, decrease the maximum amplitude of ST-segment depression⑤Decrease hs-CRP, MPO, LXA4, TNF-α, IL-1, and IL-6 levels⑥Improve coronary blood flow reserve⑩Reduce the dosage of nitroglycerin needed	16,17,19,20
			STEMI	⑥⑧Reduce myocardial no-reflow and infarction area significantly after emergency percutaneous coronary intervention for acute ST segment elevation myocardial infarction	39
Qishen Yiqi dropping pills	Huangqi (*Astragali Radix*), Danshen (*Salviae Miltiorrhizae Radix Et Rhizoma*), Sanqi (*Notoginseng Radix Et Rhizoma*), Jiangxiang (*Dalbergiae Odoriferae Lignum*)	replenishing qi and dredging the pulse, activating blood and relieving pain	Microvascular angina	②Improve the total effective rate of clinical efficacy④extend the total treadmill exercise time⑧Increase LVEF and enhance six minute walk distance	21
Naoxintong capsule	Huangqi (*Astragali Radix*), Shuizhi (*Hirudo*), Dilong (*Pheretima*), Quanxie (*Scorpio*), Danggui (*Angelicae Sinensis Radix*), Chuanxiong (*Chuanxiong Rhizoma*), Danshen (*Salviae Miltiorrhizae Radix Et Rhizoma*), Chishao (*Paeoniae Radix Rubra*), Ruxiang (*Olibanum*), Taoren (*Persicae Semen*), Honghua (*Carthami Flos*), Guizhi (*Cinnamomi Ramulus*), Sangzhi (*Mori Ramulus*), Niuxi (*Achyranthis Bidentatae Radix*), Moyao (*Myrrha*), Jixueteng (*Spatholobi Caulis*)	replenishing qi and activating blood, resolving stasis and dredging collaterals	Microvascular angina	①Reduce the frequency of angina pectoris, Shorten the duration of angina pectoris②Improve the total effective rate of clinical efficacy④Improve the treadmill exercise test⑩Reduce the dosage of nitroglycerin needed	18
Shexiang Tongxin dripping pills	Bingpian (*Borneolum*), Xiongdanfen (*Pulvis Fellis Ursi*), Niuhuang (*Bovis Calculus*), Danshen (*Salviae Miltiorrhizae Radix Et Rhizoma*), Chansu (*Bufonis Venenum*), Renshen (*Ginseng Radix Et Rhizoma*), Shexiang (*Moschus*)	replenishing qi and dredging the pulse, activating blood, resolving stasis and relieving pain	Microvascular angina	①Reduce the frequency of angina pectoris, Shorten the duration of angina pectoris②Improve the total effective rate of clinical efficacy④Extend the total treadmill exercise times, decrease the maximum amplitude of ST-segment depression⑤Decrease the level of hs-CRP⑥Improve coronary blood flow reserve⑨Improve Seattle Angina Questionnaire scores⑩Reduce the dosage of nitroglycerin needed	22,23
Shexiang Baoxin pills	Shexiang (*Moschus*), Renshen(*Ginseng Extract Et Rhizoma* ), Niuhuang (*Bovis Calculus*), Rougui (*Cinnamomi Cortex*), Suhexiang (*Styrax*), Chansu (*Bufonis Venenum*), Bingpian (*Borneolum*)	aroma warming, replenishing qi and strengthening heart	Microvascular angina	①Reduce the frequency of angina pectoris, shorten the duration of angina pectoris②Improve the total effective rate of clinical efficacy③Increase NO level and reduce ET-1 evel④Reduce the sum of exercise-induced ST segment ischemic downdraft and reduce the positive rate of treadmill exercise test⑥Increase CFR, reduce IMR, and decrease TIMI blood flow grading⑦Decrease TG, TC, and LDL-C levels and increase HDL-C level⑩Reduce the dosage of nitroglycerin needed	24,25,26,30,31,32,34
Compound Danshen Dripping pills	Danshen (*Salviae Miltiorrhizae Radix Et Rhizoma*), Sanqi (*Notoginseng Radix Et Rhizoma*), Bingpian (*Borneolum*)	activating blood and resolving stasis, regulating qi and relieving pain	Microvascular angina	①Reduce the frequency of angina pectoris, shorten the duration of angina pectoris②Improve the total effective rate of clinical efficacy	27
			No-reflow after PCI	⑥Decrease TIMI blood flow grading⑧Increase LVEF	40
Wide chest aerosol	Tanxiang (*Santali Albi Lignum*), Bibo (*Piperis Longi Fructus*), Xixin (*Asari Radix Et Rhizoma*), Gaoliangjiang (*Alpiniae Officinarum Rhizoma*), Bingpian (*Borneolum*)	regulating qi and relieving pain	Microvascular angina	①Shorten the duration of angina pectoris⑥Decrease TIMI blood flow gradeing	36
Suxiao Jiuxin pills	Chuanxiong (Chuanxiong Rhizoma), Bingpian (Borneolum)	moving qi and activating blood, resolving stasis and relieving pain	Microvascular angina	①Reduce the frequency of angina pectoris, shorten the duration of angina pectoris③Increase NO level and reduce ET-1 evel	28
XueFu Zhuyu capsule	Taoren (Persicae Semen), Chishao (Paeoniae Radix Rubra), Honghua (Carthami Flos), Danggui (Angelicae Sinensis Radix), Dihuang (Rehmanniae Radix), Chuanxiong (Chuanxiong Rhizoma) Niuxi (Achyranthis Bidentatae Radix), Jiegeng (Platycodonis Radix), Zhiqiao (Aurantii Fructus), Gancao (Glycyrrhizae Radix Et Rhizoma), Chaihu (Bupleuri Radix), Chishao (Paeoniae Radix Rubra)	activating blood and resolving stasis, moving qi and relieving pain	Microvascular angina	①Improve the total effective rate of angina pectoris②Improve the total effective rate of clinical TCM syndromes④Extend the total treadmill exercise times and reduce the sum of exercise-induced ST segment ischemic downdraft	35
Yindan Xinnaotong soft capsule	Yinxingye (*Ginkgo Folium*), Danshen (*Salviae Miltiorrhizae Radix Et Rhizoma*), Dengzhanxixin (*Erigerontis Herba*), Jiaogulan (*Herba Gynostemmatis Pentaphylli*), Shanzha (*Crataegi Fructus*), Dasuan (*Allii Sativi Bulbus*), Sanqi (*Notoginseng Radix Et Rhizoma*), Aipian (*Ɩ-Borneolum*)	activating blood and resolving stasis, moving qi and relieving pain, promoting digestion and resolving food stagnation	Microvascular angina	①Improve the total effective rate of angina pectoris, Reduce the frequency of angina pectoris, and reduce Canadian Society of Cardiology angina pectoris grade③Increase NO level and reduce ET-1 evel④Extend the total treadmill exercise times and the time of ST-segment depression of 1 mm⑤Decrease hs-CRP and IL-1β levels⑥Decrease IMR⑨Improve Seattle Angina Questionnaire scores	28, 33
Tongmai Yangxin pills	Dihuang (Rehmanniae Radix), Jixueteng (Spatholobi Caulis), Maidong (Ophiopogonis Radix), Gancao (Glycyrrhizae Radix Et Rhizoma), Heshouwu (Polygoni Multiflori Radix), Ejiao (Asini Corii Colla), Wuweizi (Schisandrae Chinensis Fructus), Dangshen (Codonopsis Radix), Guijia (Testudinis Carapax Et Plastrum), Dazao (Jujubae Fructus), Guizhi (Cinnamomi Ramulus)	replenishing qi and nourishing yin, dredging the pulse and relieving pain	Microvascular angina	①Improve the total effective rate of angina pectoris and reduce Canadian Society of Cardiology angina pectoris grade④Reduce the positive rate of treadmill exercise test	37
Danshen tablets	Danshen (Salviae Miltiorrhizae Radix Et Rhizoma)	activating blood and resolving stasis	Microvascular angina	①Improve the total effective rate of angina pectoris, reduce the frequency of angina pectoris, shorten the duration of angina pectoris, ④Enhance the time of ST-segment depression of 1 mm⑤Decrease the level of hs-CRP⑩Reduce the dosage of nitroglycerin needed	38

①Angina symptom. ②Clinical efficacy. ③Vascular endothelial function. ④Treadmill exercise test. ⑤Inflammatory indexes level. ⑥Coronary microcirculation function. ⑦Blood lipids. ⑧Cardiac function. ⑨Quality of life. ⑩The dosage of nitroglycerin needed

## Abbreviations

CMVD: Coronary microvascular disease
TCM: traditional Chinese medicine
WM: Western Medicine
CFR: coronary flow reserve
MACE: major adverse cardiovascular events
COVADIS: Coronary Vasomotion Disorders International Study Group
ESC: European Society of Cardiology
AHA: American Heart Association
MINOCA: myocardial infarction with no-obstructive coronary arteries
EAPCI: European Association of Percutaneous Cardiovascular Interventions
INOCA: Ischemia and No Obstructive Coronary Artery Disease
IMR: index of microvascular resistance
CPMs: Chinese patent medicines
PCI: percutaneous coronary intervention
CTA: coronary computed tomography angiography
OCAD: obstructive coronary artery disease
CAD: coronary artery disease
CMD: coronary microvascular dysfunction
TIMI: thrombolysis in myocardial infarction
FFR: fractional flow reserve
SPECT: single-photon emission computed tomography
MRI: magnetic resonance imaging
STEMI: ST-elevation acute myocardial infarction
LVEF: left ventricular ejection fraction
CMECs: cardiac microvascular endothelial cells
H/R: hypoxia/reoxygenation
I/R: ischemia/reperfusion
ET-1: endothelin-1
NO: nitric oxide
FMD: flow-mediated vasodilation
CRP: C-reactive protein
hs-CRP: hypersensitive C-reactive protein
AMI: acute myocardial infarction
MDA: malondialdehyde
LPO: lipid peroxide
SOD: superoxide dismutase
VEGF: vascular endothelial growth factor
bFGF: basic fibroblast growth factor
